# CroSSED sequence, a new tool for 3D processing in geosciences using the free software 3DSlicer

**DOI:** 10.1038/s41597-020-00614-y

**Published:** 2020-08-14

**Authors:** Javier Dorador, Francisco J. Rodríguez-Tovar

**Affiliations:** 1 Department of Earth Sciences, Royal Holloway University of London, Egham, UK; 2 Departamento de Estratigrafía y Paleontología, Universidad de Granada, Granada, Spain

**Keywords:** Palaeontology, Sedimentology

## Abstract

The scientific application of 3D imaging has evolved significantly over recent years. These techniques make it possible to study internal features by non-destructive analysis. Despite its potential, the development of 3D imaging in the Geosciences is behind other fields due to the high cost of commercial software and the scarce free alternatives. Most free software was designed for the Health Sciences, and the pre-settled workflows are not suited to geoscientific materials. Thus, an outstanding challenge in the Geosciences is to define workflows using free alternatives for Computed Tomography (CT) data processing, promoting data sharing, reproducibility, and the development of specific extensions. We present CroSSED, a processing sequence for 3D reconstructions of CT data, using 3DSlicer, a popular application in medical imaging. Its usefulness is exemplified in the study of burrows that have low-density contrast with respect to the host sediment. For geoscientists who have access to CT data and wish to reconstruct 3D structures, this method offers a wide range of possibilities and contributes to open-science and applied CT studies.

## Introduction

Computed Tomography (CT) techniques and other non-destructive 3D imaging tools are being widely applied in many scientific disciplines (e.g. the Health Sciences, the Arts, and the Earth Sciences) due to their non-invasive revelation of internal structures^1–3^. Within the Geosciences, CT techniques, X-ray CT and micro-CT are becoming widely used^4,5^, as reflected by the recent surge in publications^5–11^. CT enables geoscientists to study internal features (e.g. porosity, permeability, fractures, fossils, mineral composition) without destroying the sample. This greatly facilitates the study of internal rock fractures, fossils that cannot be isolated from the host sediment, museum specimens, and many other examples that would otherwise be impossible to examine.

Computed Tomography data acquisition is becoming less expensive and easier to use than some years ago. In the last few years, CT has become a standard method for collecting data from cores drilled during oceanic expeditions^12^ and their access is open to any researcher after a moratorium^13^. Many research institutions have their own CT scanner. Still, access to CT-processing software with a specific extension for the Geosciences is not common because of the high cost of commercial software licenses and only scarce free alternatives (e.g., SPIERS^14^ software), resulting in limited use of this valuable resource.

Free CT processing software^15^ —such as Drishti^16^, Fiji^17^ or 3DSlicer^18^ — was developed mainly for the Health and Life sciences. Their extensive use in recent years has led to major advances in these areas^19,20^. Moreover, specific extensions and toolkits have been developed^21,22^, 3DSlicer being the most widely used application in medical imaging^23^. It has been used in some Geoscience studies, mainly for the reconstruction of vertebrate fossils and for rock-fracture analysis^24–26^. It is unclear whether its limited use is because of technical problems owing to the density properties of materials studied in the Geosciences, or simply because geoscientists tend to use other options given a lack of specific instructions and workflows. However, the 3DSlicer could be highly useful for geoscientists working with CT data, as it has many strengths, and offers a more intuitive interface than other choices. Like other open-source software (e.g. Drishti^16^ or Fiji^17^), it allows for the development of specific toolkits, and interaction with a very active community forum. It can be run in any computer with sufficient memory and graphic capabilities, regardless of the operating system. For all these reasons, we recommend it as the most appropriate software for the study of 3D structures following the proposed workflow when density contrast is low. Free alternatives such as SPIERS^14^ may be very useful when working with data that require manual intervention, but processing is more time consuming; and Drishti^16^ is good for rendering volume from high density contrast data.

We present CroSSED as a novel processing sequence using 3DSlicer applied to Earth Science studies. For example, this sequence has been used to study bioturbation structures in marine cores, and its use could be extended to many other geoscientific disciplines. This initial step is meant to bolster the application of this free software within Earth Science studies, developing specific extensions, toolkits, and routines that will facilitate the analysis of already collected CT data.

## Results

**Fig. 1:**
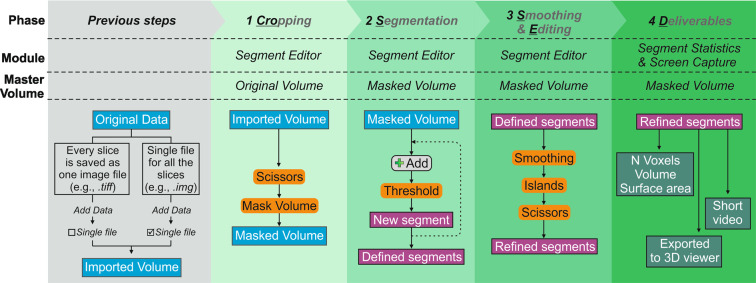
Schematic workflow of CroSSED sequence.

CroSSED offers a comprehensive sequence for volume reconstruction and 3D data analysis of discrete internal structures. Processing was done by means of 3DSlicer 4.10.2 (https://www.slicer.org/)^18^ using *.img* files, but it can be run with most file extensions generated by a CT scanner, even an image sequence (e.g. *.tiff* files). For those formats that the software cannot import, image stacks can be created, for import, using open source tools such as Drishti import^16^, or Fiji/ImageJ^17^. The proposed technique was called CroSSED as an acronym that summarizes the phases to follow: 1, *Cro*pping; 2, *S*egmentation; 3, *S*moothing & *E*diting; and 4, *D*eliverables (Fig. 1). This sequence is easy to follow, requiring no processing background or programming skills thanks to the friendlier interface of the software.

### Previous steps

First, the original data gathered by CT scanning must be imported into the software. 3D Slicer supports most extension files used by CT scanners, and all are imported in the same way (Fig. 1). The user needs only to identify whether the dataset is either a single file for all the scanned slices (e.g. *.img*, *.hdr*) or a list of images with each slice saved as an individual image file (e.g. *.tiff*, *.ima*). In the first case, the data are imported by selecting ‘Add Data’ from the ‘File’ menu and by browsing the file to add (Fig. 1). However, if the dataset is a set of images, then the user should ‘Add Data’, and select just one image file from the contain folder, then mark ‘Show options’ and unmark the ‘Single file’ option (Fig. 1). Then all the images will be uploaded in the scene view. Once this is done, the layout can be modified based on the views of interest, and many other settings can be changed. We suggest the use of ‘Four-Up’ layout and leaving the rest by default; yet if the user wishes to gather more information, they can check all the options and settings in the ‘User manual’ as well as post any question in the ‘Discussion forum’ (https://discourse.slicer.org/).

### Phase 1: Cropping

**Fig. 2:**
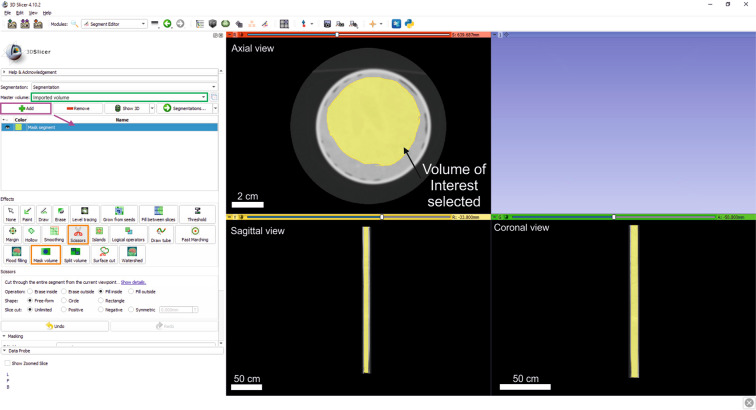
Screenshot during Cropping, selecting the volume of interest (yellow in the views). Green rectangle is the Master volume; purple rectangle points out the Add button to create new segments and orange rectangles mark the used tools (Scissors and Mask volume). Layout shows axial, sagittal, and coronal views.

Following data import, the user must select the volume of interest and remove the rest to ensure more efficient processing in subsequent phases. To this end, the user should go into the ‘Segment Editor’ module and select the original as the Master volume in which all modifications will be applied (Fig. 1). Once selected, a new segment must be created and added to the list (Fig. 2). This segment will represent the volume of interest, which is the study volume within the scanned sample. We suggest using the ‘Scissors’ tool (Figs. 1, 2), with which the user can manually define a region by selecting or erasing either the inside or outside of the shape drawn in 2D views (i.e. axial, sagittal, and coronal views). If the volume of interest is a circle or a rectangle, the shape can be chosen with the tool option and the region does not have to be drawn. In the example illustrated, as we work with sediment cores, we manually select the cylinder of sediments from the study intervals, avoiding the plastic container tube. Afterwards, this segment is cropped using the ‘Mask volume’ tool (Figs. 1, 2), filling the volume outside the segment with value 0. This process generates an output volume, after clicking on ‘Apply’; it will be called Masked volume to avoid confusion. This new volume becomes the new master volume, in which every process will be applied through the following steps of the sequence.

### Phase 2: Segmentation

**Fig. 3:**
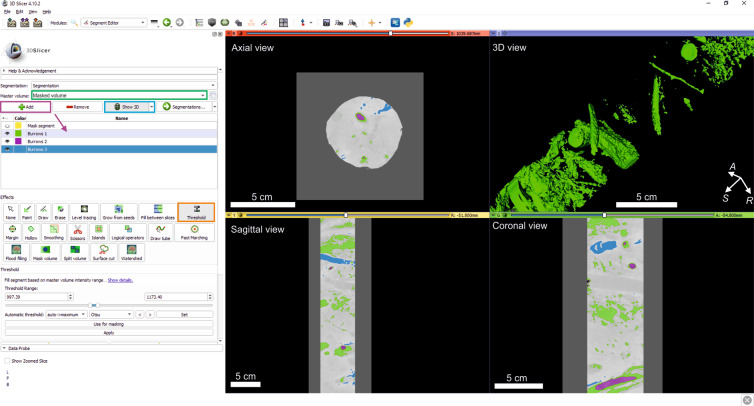
Screenshot during Segmentation, creating new segments corresponding to the study structures. The green rectangle is the Master volume; the purple rectangle points to the Add button for creating new segments; the blue rectangle points to the Show 3D button; and the orange rectangle points to the tool used (Threshold). The layout shows the axial, sagittal, coronal, and 3D views.

After establishing the volume of interest (i.e. Masked volume), the user will select it as the master volume to apply all the following steps (Fig. 3). The second phase consists of isolating the structures that will be analysed (trace fossils in our case). New segments must be defined based on the voxel values of these structures. These segments are created by clicking on the ‘Add’ button (Fig. 3). The segment colour and name can be modified simply by clicking on them, and the range of voxel values included in this new segment must be defined based on the properties of the structures studied. We suggest the use of the ‘Threshold’ tool, which defines the minimum and maximum values of the range that identifies the structures. It can be run automatically using pre-settled techniques (e.g. Otsu, Maximum entropy), although manual thresholding gives the best results. Selection can be checked in the slices shown in the 2D view windows, and once a selection fits the structures, the user need only click on ‘Apply’. This procedure can be repeated for each segment (i.e. structure) the user wishes to define (i.e. isolate) if they are represented by different parts of the CT histogram. Moreover, the user can add new segments, avoiding the previously defined ones, or even overwrite other segments.

### Phase 3: Smoothing & Editing

**Fig. 4:**
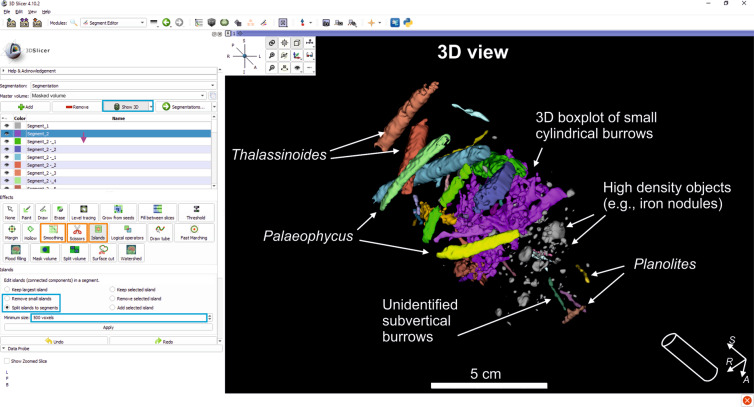
Screenshot during Smoothing and Editing, splitting edited segments corresponding to the burrows being studied. The blue rectangle points to the Show 3D button, and the orange rectangles point to the tools used (Smoothing, Scissors, and Islands) with the used functions and parameters in blue. The layout shows the 3D view.

Once segments are defined, they usually need to be edited in order to refine selected voxels by removing the noise and smoothing some contours (Fig. 4). For this task, we recommend using the ‘Smoothing’, ‘Islands’, and ‘Scissors’ tools (Figs. 1, 4). The ‘Smoothing’ tool allows smoother contours to be generated from the selected structures, controlling the strength, using either one or all the defined segments. Concretely, we applied the option ‘Joint smoothing’, which smooths all visible segments at once, but there are four other available algorithms that can be applied on individual segments: Median, that removes small details; Opening, to remove extrusions; Closing, to fill sharp corners; and Gaussian, to smooth all contours. Then, to delete the noise, the ‘Islands’ tool must be used to remove any segments smaller than the amount of voxels. This should be used carefully so as not to remove any small floating structures that one wants to keep. Finally, if further modifications are needed, we recommend the use of the operation ‘Erase inside’ from the ‘Scissors’ tool for manual removal of wrongly selected voxels, drawing an area and removing all the voxels inside.

Once the segments are smoothed, the ‘Islands’ tool can be used to split the defined segments, creating one segment per individual structure. This is useful to distinguish unconnected structures with the same voxel values. In our case for instance, we can characterize every burrow (except for overlapping structures with the same voxel values) as a different segment. In the example illustrated in Fig. 4, *Palaeophycus*, *Planolites*, *Thalassinoides*, plus some unidentified burrows, were characterized as individual segments. However, splitting was not possible in a short interval characterized by a 3D boxplot of many overlapping galleries (Fig. 4). The software offers some other editing operations that can be run, such as the tool ‘Fill between Slices’ to join unconnected burrows (e.g. burrows broken by fractures), so that the user can re-design this stage in view of the research objectives.

### Phase 4: Deliverables

**Fig. 5:**
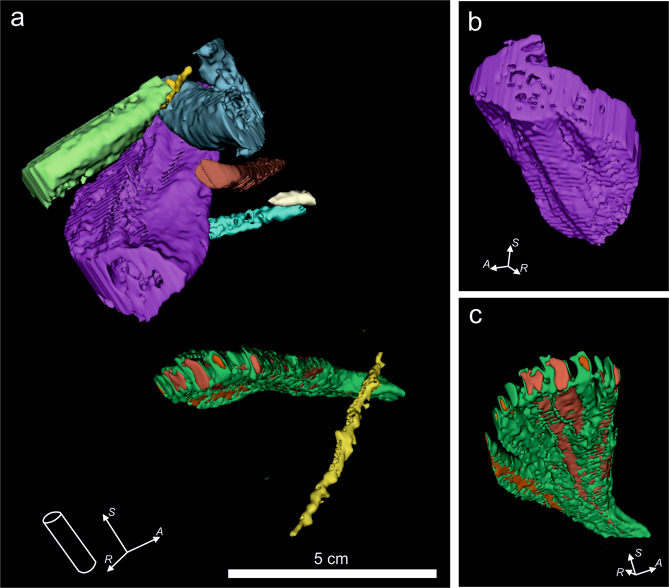
Details from some of the segmented structures after the application of CroSSED sequence. (**a)** Shows the main trace fossils isolated in the study intervals. (**b)** Shows details of the bottom part of a *Scolicia*. (**c)** Shows details from an isolated *Zoophycos* representing spreiten in different colours (green and orange).

At this point, all the structures are segmented, the noise and other artefacts have been removed, and the 3D reconstruction is displayed (Fig. 4). This is the main deliverable from processing, showing 3D structures otherwise impossible to obtain. Some further features allow us to provide a better characterization of the trace-fossil assemblage, as shown in the interval illustrated in Fig. 5. For example, some details in the bottom part of the purple burrow in Fig. 5a revealed a trilobate structure, favouring assignation to *Scolicia* (Fig. 5b). In addition, segmented structures were isolated from the green/orange burrow in Fig. 5a, supporting the attribution of spreiten *Zoophycos* (Fig. 5c). 3DSlicer provides other useful deliverables from the 3D volume, such as quantification or filming. The ‘Segment Statistics’ module gives users the opportunity to quantify certain major features from the isolated structures, e.g. the Number of voxels, Volume or Surface area (Fig. 1; Table 1). In the study case, volume was useful as it helped us to analyse the magnitude and effect of some burrows with respect to porosity, permeability, and connectivity of the material under study, this approach being of special interest for reservoir exploration.

**Table 1:** Number of voxels, Volume (mm^3^) and Surface area (mm^2^) from the isolated structures.

Segment	N voxels	Volume (mm^3^)	Surface area (mm^2^)	Volume (%)	Vol. (sum %)
High density nodules	50117	1252.93	2114.66	0.390	0.390
3D boxplot	217071	5426.78	7419.08	1.691	1.980
3D boxplot	24039	600.975	930.321	0.187	
3D boxplot	13083	327.075	565.072	0.102	
*Palaeophycus*	33763	844.075	803.813	0.263	0.590
*Palaeophycus*	23653	591.325	580.172	0.184	
*Palaeophycus*	10854	271.35	309.278	0.085	
*Palaeophycus*	7534	188.35	222.606	0.059	
*Planolites*	2189	54.725	134.047	0.017	0.094
*Planolites*	1620	40.5	94.7551	0.013	
*Planolites*	1211	30.275	65.5392	0.009	
*Planolites*	1044	26.1	68.5656	0.008	
*Planolites*	938	23.45	55.9278	0.007	
*Planolites*	829	20.725	51.5676	0.006	
*Planolites*	825	20.625	56.2561	0.006	
*Planolites*	758	18.95	47.6682	0.006	
*Planolites*	740	18.5	47.9489	0.006	
*Planolites*	724	18.1	48.3369	0.006	
*Planolites*	644	16.1	41.0657	0.005	
*Planolites*	560	14	37.8581	0.004	
*Thalassinoides*	114431	2860.78	1781.69	0.891	2.673
*Thalassinoides*	38080	952	712.835	0.297	
*Thalassinoides*	96979	2424.48	1577.28	0.755	
*Thalassinoides*	48065	1201.63	845.35	0.374	
*Thalassinoides*	45676	1141.9	918.678	0.356	
Unidentif. Burrow	4779	119.475	247.724	0.037	0.207
Unidentif. Burrow	4433	110.825	229.59	0.035	
Unidentif. Burrow	2864	71.6	155.827	0.022	
Unidentif. Burrow	2135	53.375	116.288	0.017	
Unidentif. Burrow	2046	51.15	121.875	0.016	
Unidentif. Burrow	1934	48.35	109.926	0.015	
Unidentif. Burrow	1623	40.575	79.4724	0.013	
Unidentif. Burrow	1394	34.85	83.4501	0.011	
Unidentif. Burrow	1293	32.325	71.4488	0.010	
Unidentif. Burrow	877	21.925	43.9041	0.007	
Unidentif. Burrow	798	19.95	51.1517	0.006	
Unidentif. Burrow	670	16.75	42.0606	0.005	
Unidentif. Burrow	584	14.6	36.7408	0.005	
Unidentif. Burrow	559	13.975	36.5969	0.004	
Unidentif. Burrow	557	13.925	31.2399	0.004	

The relative volume of each segment (volume %) and total (sum %) from every kind of structure were calculated taking into account that the core cylinder has a volume of 321 × 103 mm^3^ (118.2 mm in length and 58.8 mm in diameter). Then, considering studied core cylinder has a volume of 321 × 103 mm^3^ (118.2 mm length and 58.8 mm diameter) the relative volume of every segment and sum from every kind of structure has been calculated.

Another deliverable is filming in 3D analysis, a highly illustrative way to display the study structures to a general audience lacking access to specific software. The ‘Screen Capture’ module provides a short video showing the rotation of the volume determined (Fig. 1; A interval short video^27^ and B interval short video^27^). Segmentation can be exported (Segmentation >Export to file; in Segment Editor module) and then opened with any 3D visualization software (e.g. 3D Viewer in Windows), with which the user can check and adjust the volume with no further support (A interval 3D model^27^ and B interval 3D model^27^). The exported files may also be used to print clasts with a 3D printer, to be uploaded in an online virtual gallery, or for virtual reality, simply using a smartphone application for this purpose.

## Discussion

We describe a processing sequence, CroSSED, useful for 3D analysis in Geosciences, working with CT data conducted using the free and open-source software 3DSlicer. This sequence is an easy method for the 3D reconstruction, quantification, and visualization of any structure, even in cases that are difficult to differentiate because of low density contrast of the host material. As an example to evaluate the usefulness of the proposed sequence in Earth Science research, we applied this technique to the ichnological analysis of certain biogenic structures (i.e. trace fossils) from several soft sediment cores. Despite the low density contrast between the trace fossils and the host sediment, CroSSED enabled us to segment most of the discrete trace fossils, providing a 3D reconstruction and other deliverables, constituting significant progress in ichnological research involving low density contrast cases.

The application of the CroSSED sequence is advisable for any study in Geoscience and even in other disciplines apart from the Earth Sciences. This sequence has many strengths. First, CroSSED can be applied by any user regardless of their computer skills, because it is clear and easy to follow, and 3D Slicer is user-friendly software. Moreover, this software can be run in any computer, whatever the operating system, working for Windows, Mac, and Linux. Second, it is free and available for any scientist interested in 3D data, therefore enhancing networking, data sharing and the reproducibility of published results. Furthermore, specific modules, extensions or tools can be developed, since it is open-source software. Third, because it is not an automated process or tool, CroSSED can be applied regardless of the density of the study material, even in cases where the density contrast between the study structure and the host material is relatively low. Finally, the proposed processing sequence is useful not only for 3D visual inspection, but also for quantitative analyses, such as volume or surface data.

On the other hand, certain weaknesses should be considered. Currently, CT data acquisition is not as difficult and expensive as it was some years ago, and there are even some open repositories, so it is no longer a general limitation. Yet CT processing requires certain minimum hardware requirements in terms of graphic capabilities and memory (i.e. 1GB Dedicated graphics hardware and 8 GB memory are recommended by the developers of 3DSlicer). In such cases, when processing takes too long, we recommend masking a smaller volume during *Phase 1: Cropping* to decrease the volume of data to be processed, or else downsampling the data before processing. Furthermore, although CroSSED can be applied in materials of low density contrast, and the threshold parameters are controlled by the user, it requires some minimal contrast to isolate the structures. In samples with very low density contrast, segmentation of the objects might entail including too much noise, calling for more smoothing, or it may be impossible to segment them in the worst cases. This is common for all threshold-based approaches and software; in some cases such limitations can be reduced by pre-processing and filtering the data before importing them. With respect to quantitative analyses, it should be noted that some quantitative data might be affected by previous systematic errors (during processing) owing to technical limitations (e.g. noise, data resolution, chosen parameters). For all these reasons, we consider that the processing sequence expounded here stands as a first step forward. Eventually the software can be applied to other scientific disciplines, with specific routines and extensions created to facilitate the analysis of CT data and promote data sharing and reproducibility. In the examples presented here —the study of bioturbation structures, having both scientific and economic implications— CroSSED serves to gather information on ichnotaxonomy, ichnodiversity, and other ichnological features of interest for sedimentary basin analysis, but also on permeability, porosity, and connectivity, of interest in reservoir exploitation. In addition to proving useful for research, it can be used to disseminate results to the general public^28,29^. Short videos can be generated and 3D files can be exported to be visualized using a common software, or printed using a 3D printer, or even viewed as virtual reality using a smartphone application.

## Methods

The proposed method was checked on 15 soft sediment cores from marine settings. Specifically, 13 of them were downloaded from the Virtual Core Library of samples drilled by D/V CHIKYU^13^ (316-C0006F-12R-2, 322-C0012A-34R-1, 322-C0012A-34R-2, 333-C0011C-1H-1, 333-C0011C-1H-2, 333-C0011C-1H-5, 333-C0011C-2H-1, 333-C0011C-2H-2, 333-C0011D-3H-6, 333-C0011D-40X-6, 333-C0011D-41X-3, 333-C0011D-41X-4, 333-C0011D-49X-4) and two of them from gravity cores collected during ForSaGal 09^30,31^ (FSG09-07, FSG09-17). Cores drilled by D/V CHIKYU were scanned immediately after core cutting using an X-ray CT scanner (LightSpeed Ultra 16, GE Yokogawa Medical Sys-tems, Ltd.) on the ship. Two other cores were scanned at the Veterinary Teaching Hospital Rof Codina in Lugo (Spain) using a multi-slice CT scanner (HITACHI ECLOS 16).

Processing was conducted on 3D Slicer 4.10.2 (https://www.slicer.org/)^18^ after previous installation of SegmentEditorExtraEffects extension (http://slicer.kitware.com/midas3/slicerappstore/extension/view?extensionId=293582) from the Extension Manager. All the cores were divided in approximately 50 cm intervals to make the processing easier and faster. Specifically, in this contribution we show some illustrative intervals cropped from 316-C0006F-12R-2, 322-C0012A-34R-1 and FSG09_07 cores.

## Data Availability

The CT data files of samples drilled by D/V CHIKYU are available in the Virtual Core Library (http://www.kochi-core.jp/VCL/xCTdata.html). Supplementary files and a worked up dataset as example (Processed example) have been uploaded to figshare^27^.

## Code availability

3D Slicer is available at https://www.slicer.org/. All the content is available under Creative Commons Attribution-ShareAlike. The source code of the application itself is available at https://github.com/Slicer/Slicer.
